# *Anopheles gambiae* Trehalase Inhibitors for Malaria Vector Control: A Molecular Docking and Molecular Dynamics Study

**DOI:** 10.3390/insects13111070

**Published:** 2022-11-19

**Authors:** Eunice O. Adedeji, Gbolahan O. Oduselu, Olubanke O. Ogunlana, Segun Fatumo, Rainer Koenig, Ezekiel Adebiyi

**Affiliations:** 1 Covenant University Bioinformatics Research (CUBRe), Covenant University, Ota 112233, Nigeria; 2 Department of Biochemistry, College of Science and Technology, Covenant University, Ota 112233, Nigeria; 3 Department of Chemistry, College of Science and Technology, Covenant University, Ota 112233, Nigeria; 4 Covenant Applied Informatics and Communication Africa Centre of Excellence (CApIC-ACE), Covenant University, Ota 112233, Nigeria; 5 Department of Non-Communicable Disease Epidemiology, London School of Hygiene & Tropical Medicine, London WC1H 9SH, UK; 6 Institute for Infectious Diseases and Infection Control (IIMK, RG Systemsbiology), Jena University Hospital, Am Klinikum 1, 07747 Jena, Germany; 7 Department of Computer and Information Sciences, College of Science and Technology, Covenant University, Ota 112233, Nigeria; 8 Division of Applied Bioinformatics, German Cancer Research Center (DKFZ), G200, Im Neuenheimer Feld 280, 69120 Heidelberg, Germany

**Keywords:** trehalase, trehazolin, mosquito, vector control, validamycin, validoxylamine, uniflorine, casuarine

## Abstract

**Simple Summary:**

*Anopheles gambiae* is a major malaria vector. This vector is controlled mainly using insecticide, thereby reducing malaria transmission. Since many insecticides are toxic to non-target species, and there is increasing insecticide resistance, there is a need to identify other safer molecules that can be used to control the vector. Molecules inhibiting trehalase have been proposed as safer options for insecticide and fungicide development since humans do not produce trehalose. This work screens several trehalase inhibitors against trehalase of *Anopheles gambiae* (*Ag*Tre) and assesses their safety in humans using in silico methods. Four trehalase inhibitors had a high affinity for *Ag*Tre and were predicted to be relatively safe. The compounds also interacted well with trehalase of *Aedes aegypti*, another important mosquito vector. The results suggest these molecules are safer options for insecticide development to control mosquito-borne diseases.

**Abstract:**

Trehalase inhibitors are considered safe alternatives for insecticides and fungicides. However, there are no studies testing these compounds on *Anopheles gambiae,* a major vector of human malaria. This study predicted the three-dimensional structure of *Anopheles gambiae* trehalase (*Ag*Tre) and identified potential inhibitors using molecular docking and molecular dynamics methods. Robetta server, C-I-TASSER, and I-TASSER were used to predict the protein structure, while the structural assessment was carried out using SWISS-MODEL, ERRAT, and VERIFY3D. Molecular docking and screening of 3022 compounds was carried out using AutoDock Vina in PyRx, and MD simulation was carried out using NAMD. The Robetta model outperformed all other models and was used for docking and simulation studies. After a post-screening analysis and ADMET studies, uniflorine, 67837201, 10406567, and Compound **2** were considered the best hits with binding energies of −6.9, −8.9, −9, and −8.4 kcal/mol, respectively, better than validamycin A standard (−5.4 kcal/mol). These four compounds were predicted to have no eco-toxicity, Brenk, or PAINS alerts. Similarly, they were predicted to be non-mutagenic, carcinogenic, or hepatoxic. 67837201, 10406567, and Compound **2** showed excellent stability during simulation. The study highlights uniflorine, 67837201, 10406567, and Compound **2** as good inhibitors of *Ag*Tre and possible compounds for malaria vector control.

## 1. Introduction

Mosquitoes such as *Anopheles* and *Aedes* spp. are important vectors of several human diseases [1]. These mosquito-borne diseases, such as malaria, are controlled principally by employing vector-control strategies [2]. These strategies aim to reduce or eliminate the contact of the vector with humans, thus preventing parasite transmission. The primary vector control strategy for malaria involves the use of insecticides either in insecticide-treated nets (ITNs) or for indoor residual spraying (IRS) [3]. These strategies have contributed considerably to averting clinical cases of the disease, with about sixty-eight per cent of the 663 million averted cases between 2000 and 2015 attributed to the use of ITNs [4]. This highlights the importance of vector control in curbing malaria. However, increasing reports of insecticide resistance, the toxicity of insecticides to non-target species, and the persistence of insecticides in the environment have necessitated the search for safer alternatives in vector control [5,6]. Larval source management (LSM) strategies, which prevent the emergence of adult mosquitoes, are one of the complementary strategies employed in vector control [7]. These methods reduce the number of house-entering as well as outdoor biting mosquitoes and have been observed to reduce malaria transmission when combined with the use of ITNs [8]. Amidst this, increasing research efforts are being made to identify and develop new classes of safer compounds that could be used in vector control.

Trehalase inhibitors have been proposed as safer alternatives for insecticides as they are non-toxic and do not persist in the environment [9]. Trehalase (EC 3.2.1.28) is an enzyme that catalyses the reduction of trehalose into two molecules of glucose [10,11]. This reaction is critical in insects, as trehalose is the major hemolymph sugar in insects and is also the fuel required for flight [12]. Trehalose is also needed in larvae to resist stress factors [13]. Trehalase levels have been reported to be upregulated in the salivary gland of *Anopheles stephensi* during *Plasmodium vivax* infection, suggesting that it might also play a crucial role in parasite development in the mosquito [14]. Similarly, inhibition of trehalose transporter in *Anopheles gambiae* was observed to lead to decreased survival of the mosquito as well as decreased *Plasmodium* development in the mosquito [15]. Inhibition of trehalase by validamycin has been proposed as promising for the development of new insecticides [16]. Validamycin A has been tested in different insect species. For example, it has been observed to cause developmental defects and decrease fecundity in *Helicoverpa armigera* [17] and cause mortality in *Diaphorina citri* [18]. Marten et al. [19] reported that validamycin A, a trehalase inhibitor, reduced egg hatching and pupation, skewed the sex ratio, and prevented flight in *Aedes aegypti*, the vector for dengue fever, chikungunya, Zika fever, Mayaro, and yellow fever. Minimal larva death in *Ae. aegypti* (7% mortality at 100 and 200 ppm, and 9% mortality at 500 ppm validamycin A administration) was observed in their study. An earlier report by Logan [20] revealed that validoxylamine A, another trehalase inhibitor, did not cause significant mortality of *Ae. Aegypti* larvae but prevented flight of emerging adults and decreased the number of eggs laid by adult *Ae. aegypti* mosquitoes. This suggests that trehalase inhibitors can serve as insect growth regulators, sterilants, and flight inhibitors. This is important as the flight of disease vectors to access humans is required for disease transmission. Similarly, sterilants can help to reduce mosquito populations. There is growing research on synthesising other potential trehalase inhibitors and testing their activities in different species as fungicides or insecticides [9]. Although these potential inhibitors have been tested in different organisms, no study has tested these compounds in the malaria vector, *Anopheles* spp., to elucidate their possible use for malaria vector control. While validamycin A and validoxylamine A have been tested in *Ae. aegypti,* validamycin A is considered a weaker binder of trehalase, and the safety of validoxylamine A is of concern, since it is a good binder of porcine trehalase. Hence, there is a need to identify more potent and safe inhibitors of trehalase aside from validamycin A in these disease vectors, which can be developed for vector control. These can be carried out using traditional drug design methods or computer-aided drug design methods (CADD).

Traditional drug design methods are costly as they involve the synthesis of numerous compounds followed by in vitro or in vivo testing of their activities. For example, D’Adamio et al. [13] synthesised more than 20 compounds and tested their activity against *Chironomus riparius* trehalase. Apart from the cost involved in synthesising numerous compounds, in vitro or in vivo testing of compounds depend on the availability of recombinant enzymes or the organisms, which come with a cost that multiplies with an increasing number of compounds. However, CADD methods, which include molecular docking and molecular dynamics (MD) simulation, offer cheaper and faster means of screening and identifying potential binders of a protein from a wide library of compounds [21]. These methods rely on the availability of the three-dimensional (3D) structure of the protein of interest and a compound library [22]. Protein structure can be experimentally determined or computationally derived [23]. Potential hits, obtained based on docking scores from molecular docking, can be optimised and re-screened, leading to the guided synthesis of compounds. This helps to reduce the costs involved in insecticide/drug development. Performing molecular dynamics simulation after molecular docking helps to reveal the stability of protein–ligand interactions and may separate false positive from true positive hits [21]. As described above, trehalase inhibitors are promising vector-control compounds. Unfortunately, there is no report about their use against *An. gambiae,* an important malaria vector. In this study, the 3D-structure of *An. gambiae* trehalase (*Ag*Tre) was determined and molecular docking was employed to screen a library of compounds in order to identify potential lead structures against *Ag*Tre that could be developed for malaria vector control. Considerations were given to the interaction of these compounds to trehalase of *Ae. aegypti*, which is also an important mosquito vector of human diseases. Since trehalase from both organisms share >70% sequence identity, inhibitors could be used to control both vectors, preventing transmission of other mosquito-borne diseases aside from malaria, e.g., dengue fever. In addition, the medicinal chemistry and toxicity potential of these compounds were predicted, and the stability of their complexes with trehalase was determined. Four trehalase inhibitors were observed to be better binders of trehalase of *An. gambiae* and *Ae. aegypti* than validamycin A. The compounds were predicted to be non-toxic when compared to validoxylamine. Hence, the study proposes these inhibitors as potential molecules for vector control. 

## 2. Materials and Methods

### 2.1. Prediction of 3D Structure of AgTre

Most insects have at least two copies of trehalase genes; however, it has been reported that dipteran insects, such as *Anopheles* spp., have only one copy of trehalase [24]. The single copy gene of trehalase in *An. gambiae* is AGAP012053, which codes for its trehalase protein *Ag*Tre. The experimental 3D structure of *Ag*Tre has not been determined; hence it is not available in the database of the Research Collaboratory for Structural Bioinformatics Protein Data Bank (RCSB PDB) [25]. However, a predicted structure based on homology modelling is available in the SWISS-MODEL Repository [26]. The structure was built using trehalase of *Arabidopsis thaliana* (7E9X_A) as a template which has the highest percentage identity (33%) to *Ag*Tre. Considering the low percentage identity and family distance of the two proteins (plant to insect), ab initio modelling was implemented in predicting the 3D structure of *Ag*Tre. The FastA file containing the protein sequence of the *Ag*Tre (Trehalase from *An. gambiae* Pest strain) was retrieved from UniProt Knowledgebase (UniProtKB) [27] with the accession number Q7PZS4. The protein sequence of *Ag*Tre was submitted to three ab initio modelling web servers: Iterative Threading Assembly Refinement (I-TASSER), Contact-guided Iterative Threading ASSEmbly Refinement (C-I-TASSER) [28,29], and the Robetta web server. On the Robetta server, a deep learning-based modelling method, RoseTTAFold [30], was used to predict the 3D structure of *Ag*Tre. RoseTTAFold gives confidence scores for models (ranging from 0 to 1), with models closer to 1 being better structures. This confidence score corresponds to predicted IDDT score using DeepAccNet [31]. For I-TASSER and C-I-TASSER, default parameters were used in predicting the 3D structure for *Ag*Tre. I-TASSER and C-I-TASSER output C-scores within the range between −5 and 2, with a higher C-score signifying higher model confidence.

### 2.2. Structure Assessment of AgTre Models

Structure assessment of all predicted models in this study (RoseTTAFold, I-Tasser, and C-I-Tasser), as well as available models from SWISS-MODEL and AlphaFold, was carried out using the SWISS-MODEL web server structure assessment tool [32]. Protein model structures from AlphaFold predictions have been integrated into the SWISS-MODEL web server [33,34]. For structural assessment, Qualitative Model Energy Analysis (QMEAN) [35] values around 0 showed reliability, while values ≤−4.0 indicated a low-quality model [36]. A normalised QMEAN4 score compares a predicted model structure with a non-redundant set of PDB structures, providing information on how many standard deviations away from the mean is the predicted model score given a score distribution from a large set of experimentally determined structures. The value varies from |Z-score| < 1 to |Z-score| > 2, with |Z-score| < 1 being closer to the mean, hence a better structure. Ramachandran plot and statistics were obtained, with a model having >98% of its residues in the most favoured regions considered to be of good quality. Similarly, Ramachandran outliers should be <0.2%, Rotamer outliers <1%, number of C-Beta deviations and twisted prolines/non-prolines should be zero. Further assessment was carried out using ERRAT [37] and VERIFY3D [38,39] on SAVES servers (v6.0). For VERIFY3D and ERRAT, models with ≥80% of its amino acid residues having average 3D/1D profile scores ≥ 0.2 and overall quality factor >90%, respectively, were considered to be of good quality.

### 2.3. Active Site Prediction

The active site residues necessary for the catalytic activity of *Ag*Tre were predicted using CASTp [40] and PrankWeb servers [41]. Active site prediction for trehalase from *Ae. aegypti* (AlphaFold model of Q16V81: AF-Q16V81-F1) was carried out using PrankWeb.

### 2.4. Ligand Preparation

Validamycin A, trehalozin, trehazolamine, validoxylamine A, casuarine, and uniflorine have been reported to be inhibitors of trehalase in different species. Hence, they were used as the starting compounds to generate more similar structures [9]. However, validamycin A was employed as the control ligand or reference standard due to its commercial availability and reported activity in different insect species. Compounds with similar structures to the control ligand and the previously reported trehalase inhibitors in different species were searched for and retrieved with a 100% search of the PubChem database [42] using a Tanimoto threshold of 80%. This search resulted in a total of 3022 compounds, which were downloaded in their SDF formats. Using the OpenBabel package in PyRx software, the SDF files were minimised using the Universal Force Field (uff) [43] to obtain appropriate proper bond lengths between the different atoms. They were then converted into the pdbqt format and further used for the virtual screening studies.

### 2.5. Protein Preparation

The best modelled protein structure of *Ag*Tre and the downloaded structure of *Ae. aegypti* (AlphaFold: AF-Q16V81-F1) were prepared using UCSF Chimera [44]. The proteins were minimised using 2000 steepest descent steps and 500 conjugate gradient steps with a conjugate gradient step size of 0.02. AMBER ff14SB charges were added to standard amino acid residues. The prepared PDB file was converted to pdbqt using AutoDock in PyRx.

### 2.6. Virtual Screening

Virtual screening of the 3022 compounds against *Ag*Tre was carried out using AutoDock Vina wizard in PyRx [45]. The vina search space was fitted around the predicted active site residues leading to xyz dimensions of 28.6748, 29.3685, 26.8692, and xyz centre of 0.4168, 7.6228, −20.4605 Å. An exhaustiveness of 8 was used. Similarly, virtual screening of the starting compounds, top hits (obtained from *Ag*Tre studies), and optimised compounds (obtained from *Ag*Tre studies) against trehalase of *Ae. aegypti* (*Aa*Tre) was carried out. The binding pocket of *Aa*Tre was set at xyz dimensions of 25.8307, 27.2200, 30.0916, and xyz centre of −2.1668, −2.0597, and −6.4698 Å. When the virtual screening was completed, binding energies were obtained for the starting compounds, control ligand, and top 9 ligands. Discovery Studio was used to visualise the Trehalase—ligand binding interaction [46].

### 2.7. Toxicity Screening and Ligand Optimisation

The control ligand, previously reported inhibitors, and top 9 hits were screened for possible ecotoxic effects using admetSAR 2.0 [47], toxicity using ADMETlab 2.0 [48], and their medicinal chemistry properties were determined using SwissADME [49]. ADMETopt [50] was used to optimise the scaffolds of some top hits with the aim of seeing if optimised compounds would have a better binding affinity. A non-toxic constraint (i.e., compounds showing no carcinogenicity, Ames toxicity, acute oral toxicity) was used for optimisation. The new compounds were docked against *Ag*Tre and *Aa*Tre.

### 2.8. Molecular Dynamics Simulation

The MD simulation was carried out by using Nanoscale Molecular Dynamics (NAMD) [51] and Visual Molecular Dynamics (VMD) [52]. This helped to determine the stability of the best poses of some of the best hits from the docking studies in the binding site of *Ag*Tre. Energy minimisation of 1000 steps was performed to fix the backbone atoms, while the production simulation run was carried out for 1 ns, equivalent to 500,000 steps. The simulation was performed at a constant pressure of 1 atm and temperature of 310 K using Periodic Boundary conditions. The protein structure file (PSF) of the target was generated separately from that of the ligand using VMD, while those of ligands were generated using the Charmm36 forcefield of the Charmm-GUI web server [53]. These were generated to define the bond types, bond angles, atom types, and the number of molecules in the simulation system. The topologies (PDB and PSF) of the protein and ligands were merged, and the complexes were solvated using VMD to generate the cubic water box. The other necessary parameters (time and Periodic Boundary conditions) for the simulation were defined in a script and run using NAMD. The RMSD (Root Mean Square Deviation), RMSF (Root Mean Square Fluctuation), and PCA (Principal Component Analysis) of the simulation results were determined using Bio3D on the Galaxy Europe platform [54], while the hydrogen bond (h-bond) analysis was carried out using VMD software. Graphs were plotted using the plot() function in R-programming software [55]. 

## 3. Results

### 3.1. Structure Prediction and Assessment of AgTre

**Figure 1:**
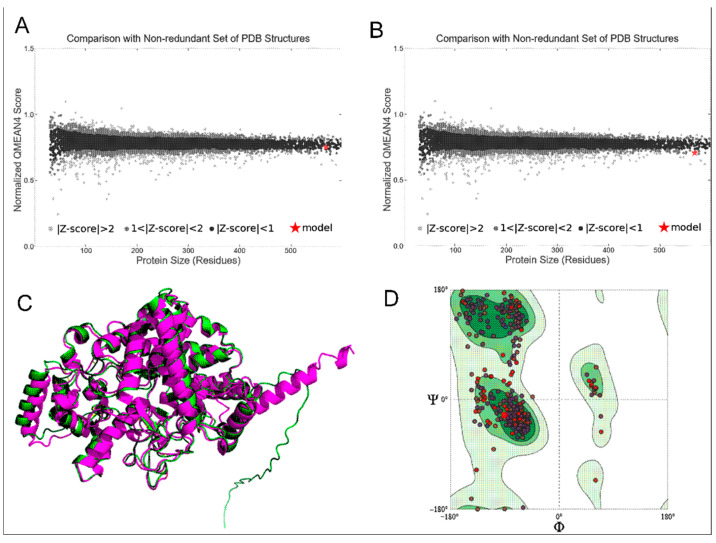
(**A**) Normalised QMEAN score of *Ag*Tre structure predicted from RoseTTAFold; (**B**) normalised QMEAN score of *Ag*Tre structure predicted by AlphaFold; (**C**) predicted 3D structure of *Ag*Tre. Green: predicted structure from AlphaFold. Purple: predicted structure using RoseTTAFold. RMSD of 0.896 Å considering 476/570 residues; (**D**) Ramachandran plot of the *Ag*Tre structure predicted using RoseTTAFold showing 98.06% of residues in Ramachandran favoured region. *Ag*Tre: trehalase of *An. gambiae*.

The 3D structure of *AgTre* predicted by RoseTTAFold had a confidence score of 0.85, which is close to 1, suggesting a good structure. The best model from I-TASSER had a C-score of −0.22, while that from C-I-TASSER had a C-score of 0.45, suggesting that the model obtained from C-I-TASSER was a better model than that obtained from I-TASSER. I-TASSER and C-I-TASSER models had QMEAN < −4.0, while other models were >−4.0. However, the RoseTTAFold model (−0.10) had QMEAN closest to 0, suggesting it to be of better quality (Table 1). Similarly, it had a normalised QMEAN4 of |Z-score| < 1 as compared to the AlphaFold model with 1 < |Z-score| < 2 (Figure 1A,B). This further suggests that the RoseTTAFold predicted model is the best comparable model with a non-redundant set of PDB structures. The predicted structure from AlphaFold had 96.13% of its residues in Ramachandran favoured, 0.70% Ramachandran outliers, and 0.82% Rotamer outliers compared to 98.06%, 0.35%, and 0%, respectively, observed for the predicted structure from RoseTTAFold in this current study (Table 1). When the RoseTTAFold model and AlphaFold model are aligned, the RMSD, which gives the average deviation between the corresponding atoms between the two proteins, was 0.896 Å when considering 476/570 residues, suggesting the two proteins had similar folds for most parts (Figure 1C). Hence, the structure in this study is comparable to that predicted using AlphaFold. Among all the models tested, the model from RoseTTAFold prediction had the highest number of residues in the Ramachandran favoured region (98.06%). It was the only model with >98% residues in the Ramachandran favoured region (Figure 1D). Based on this parameter, the models from I-TASSER (77.18%) and C-I-TASSER (79.67%) were inferior to all other models. While all models passed ERRAT and VERIFY3D, the RoseTTAFold model had no twisted prolines, cis prolines, rotamer outliers, and C beta deviations as compared to the other models (Table 1). Hence the RoseTTAFold model was observed to be the best model and was used subsequently in this study. 

### 3.2. Active Site Prediction

The predicted active site residues of the RoseTTAFold *Ag*Tre model and the *Aa*Tre model are presented in Table 2. Active site prediction from CASTp yielded a total of 116 pockets, while the prediction from PrankWeb gave 12 pockets for *Ag*Tre and 5 pockets for *Aa*Tre. The pocket with the highest probability and pocket score was selected for PrankWeb, while the pocket with the largest area and volume was selected for CASTp. The probability for the selected active site pocket for *Ag*Tre and *Aa*Tre predicted using PrankWeb were 0.948 and 0.855, respectively. For *Ag*Tre, 17 amino acids were predicted to be necessary for catalytic activity in the selected pockets from both CASTp and PrankWeb. Four additional residues were predicted to be active site residues by CASTp only. Since the predicted binding pocket from the two programs overlapped, the predicted active site residues from CASTp were used to define the grid box used for molecular docking. Twenty-one (21) residues were predicted as active site residues of *Aa*Tre. In the two species, 19 predicted active site residues of trehalase were conserved. This suggests the possibility of both organisms interacting with similar compounds.

### 3.3. Molecular Docking

The results obtained from the virtual screening and post-screening studies, ADMET studies, and lead optimisation using ADMETopt are presented in this subsection. Additionally, results comparing binding energies (obtained from virtual screening) between *Ag*Tre and *Aa*Tre are reported.

#### 3.3.1. Virtual Screening and Post-Screening Studies

The virtual screening studies revealed that all the previously reported trehalase inhibitors that were screened had lower binding energy (i.e., higher affinity) than the control ligand, validamycin A (−5.4 kcal/mol, indicating lowest affinity) for *Ag*Tre (Table 3). Among the previously reported trehalase inhibitors, validoxylamine A had the lowest binding energy (−8.8 kcal/mol), indicating the highest affinity for *Ag*Tre compared to validamycin A, followed by trehazolin (−7.5 kcal/mol). Uniflorine, casuarine, and trehazolamine had quite similar binding energies for *Ag*Tre (−6.9, −6.6, and −6.4 kcal/mol), suggesting that *Ag*Tre might have similar affinity for these compounds. Of all the 3022 compounds screened, only 9 compounds had binding energies <−8.8 kcal/mol observed for validoxylamine A (the previously reported trehalase inhibitor with the lowest binding energy). The binding energies were between −8.9 to −9.1 kcal/mol (Table 3). The compounds interacted with different residues in the active site of *Ag*Tre through conventional hydrogen bonds, carbon-hydrogen bonds, Pi interactions, and van der Waals interactions. The ligands formed conventional hydrogen bonds, Pi-donor hydrogen bonds, or carbon-hydrogen bonds with two or more of the active site residues Glu258, Asn186, Gln197, Asp292, Ser259, Gly290, Gln438, Arg256, Arg142, Tyr192, Ala287, Phe143, Trp149, Lys26. However, the control ligand, validamycin A, did not bind to any predicted active site residue of the protein. Validamycin A rather formed hydrogen bonds with Gly504, Gln506, Asn307, Ser266 (Table 3, Figures S1 and S2). Other ligands further interacted with other active site residues through van der Waal interactions. Validoxylamine, trehazolamine, 10690241, and 67837201 formed Pi-Sigma interactions with Tyr507, while 25023458 formed Pi-Sigma interactions with Phe143. In addition, validoxylamine, 101104782, 13364642, 25023458, 21021639, and 58618560 had Alkyl/Pi-Alkyl interactions with one or more of Tyr147, Phe143, Arg142, Tyr507, or Lys26. However, the only interaction validamycin A had with an active site residue was a van der Waal interaction with Tyr507. This further indicates that validamycin is a poor binder of *Ag*Tre.

#### 3.3.2. ADMET Studies and Lead Optimisation Using ADMETopt

ADME screening of the top nine hit compounds, control ligand, and previously reported inhibitors using SwissADME revealed that they were not blood–brain barrier permeants or CYP 450 inhibitors. The compounds were predicted to be soluble and had no structural alert for Pan-Assay INterference compoundS (PAINS), suggesting that they are not promiscuous binders but would be target-specific [56,57]. Of the top 9 hit compounds, only 10406567, 10690241, and 67837201 had no structural alert for Brenk (Table 4), suggesting that they are non-toxic and metabolically stable [58]. All hit compounds had synthetic accessibility scores lower than validamycin A, which had the highest synthetic accessibility score of 6.34. This suggests that these compounds might be easier to synthesise than validamycin A. The control ligand, starting compounds, and top 9 hits were predicted by admetSAR to be non-toxic to honeybees, crustaceans, and fish. The compounds were predicted to be biodegradable except casuarine and trehazolamine, which had a mild non-biodegradable probability of 0.575 and 0.55, respectively (Table 4). Likewise, employing ADMETlab, all screened compounds were predicted to be non-carcinogenic, cause no irritation or corrosion to the eyes, and cause no skin sensitisation. Predictions by ADMETlab revealed that only 10406567, 10690241, and 67837201 among the top 9 hits had probabilities <0.35 for DILI and respiratory toxicity. Among these three compounds, 10406567 and 10690241 were predicted to have a low probability of being nephrotoxic by admetSAR (probability of 0.4811 and 0.5058, respectively), while 67837201 was not nephrotoxic. Uniflorine (9794258) was predicted to be the safest previously reported compound.

Further optimisation of the scaffolds of 101104782, 10406567, 10690241, and 67837201 using ADMETopt yielded 120 compounds. These were screened further to select non-toxic compounds based on Brenk alert, a low DILI, and respiratory toxicity probability, yielding 30 compounds. These 30 compounds were docked against *Ag*Tre. The best binder was 2-{[6,7-dihydroxy-5-(hydroxymethyl)-3aH,5H,6H,7H,7aH-pyrano[2,3-d][1,3]oxazol-2-yl]amino}-6-(hydroxymethyl)oxane-3,4,5-triol (Compound **1**) with a binding energy of −8.9 kcal/mol (Table 5, Figure S3) similar to 67837201, and was predicted not to be nephrotoxic (Table 4). 67837201 and Compound **1** were observed to be the safest compounds with the lowest binding energies for *Ag*Tre. Among the optimised compounds, the next top binder after Compound **1** was 4-(hydroxymethyl)-6-{[3,4,5-trihydroxy-6-(hydroxymethyl)oxan-2-yl]amino}-hexahydro-[1,3]dioxolo [4,5-c]pyran-2-one (Compound **2**) with a binding energy of −8.4 kcal/mol (Table 5), which was predicted to be relatively non-toxic except for a predicted probability of 0.5908 for nephrotoxicity (Table 4).

#### 3.3.3. Comparison of Results from AgTre with AaTre

The control compound, previously reported trehalase inhibitors, 101104782, 10406567, 10690241, 67837201, and the 30 optimised compounds were screened against *Aa*Tre. The binding energies of these compounds in *Aa*Tre and *Ag*Tre are presented in Table 6. Similar to the trend observed with *Ag*Tre, validoxylamine A had the lowest binding energy (−8.8 kcal/mol) for *Aa*Tre, while validamycin A had the highest binding energy (−6.4 kcal/mol). Uniflorine, the safest previously reported trehalase inhibitor, had a binding energy of −7.6 kcal/mol for *Aa*Tre and −6.9 kcal/mol for *Ag*Tre. The best hit compounds for *Aa*Tre were Compound **2** and 10406567 having binding energies of −8.4 and −8.3 kcal/mol, respectively. These compounds had binding energies of −8.4 and 9.0 kcal/mol, respectively, with *Ag*Tre. Although Compound **2** and 10406567 were considered safe based on predicted ADMET parameters, they had a slight probability of being nephrotoxic (0.4811 and 0.5908, respectively) (Table 4). The safest compounds (67837201 and Compound **1**) with the lowest binding energy for *Ag*Tre (−8.9 kcal/mol) had higher binding energy for *Aa*Tre (−5.9 and −6.4 kcal/mol, respectively), suggesting they might have a lower affinity for *Aa*Tre.

### 3.4. Molecular Dynamics Simulation

The control compound (validamycin A), the previously reported trehalase inhibitor with the best affinity for trehalase (validoxylamine A), as well as 67837201, Compound **1**, Compound **2**, and 10406567 in complex with *Ag*Tre, were subjected to MD simulation.

#### 3.4.1. Root Mean Square Deviation (RMSD) and Root Mean Square Fluctuation (RMSF)

**Figure 2:**
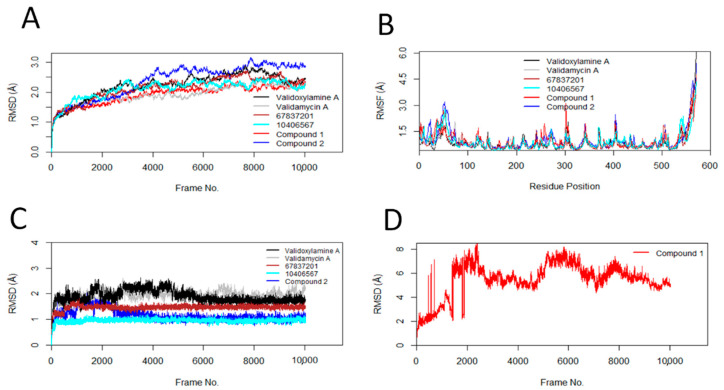
Conformational changes, residue flexibilities of the protein, and conformational changes of the ligand. (**A**) Protein Cα-RMSDs during MD simulation with ligand; (**B**) protein Cα-RMSFs; (**C**) ligand RMSDs; (**D**) RMSD of Compound **1**.

RMSD and RMSF results from the molecular dynamics simulation studies are shown in Figure 2A–D, and the individual results are in Figures S4–S8. The stability of protein, indicated by the RMSD changes with respect to Frame No, is presented in Figure 2A. Similarly, the average RMSDs of the protein and standard deviations are shown in Table 7. The binding of validamycin A, 67837201, 10406567, and Compound **1** to *Ag*Tre resulted in smaller RMSD changes in the protein compared to the binding of validoxylamine A (the previously reported trehalase inhibitor with the best affinity for trehalase). On the contrary, the binding of Compound **2** resulted in more conformational changes to the protein structure. However, all conformation changes were less than 3 Å, suggesting that the conformation of the protein was stable throughout the MD simulation [59]. RMSF describes the flexibility of each amino acid residue in the protein during the MD simulation. The larger the RMSF value is, the more flexible the residue is. Regarding Figure 2B, the amino acid residues with RMSF > 1.5 Å represent the flexible residues in the loop region of the protein. Similar flexible patterns in the protein were observed upon binding of the different ligands. The RMSDs of the ligands (validamycin A, 67837201, 10406567, Compound **2**, and validoxylamine A) are shown in Figure 2C, while that of Compound **1** is shown in Figure 2D. Similarly, the average RMSDs of the ligands and standard deviations are shown in Table 7. The average RMSDs of validoxylamine A, validamycin A, 67837201, and 10406567 reveal that these compounds do not significantly change their orientation during the simulation; hence, they form stable complexes with the protein. This is also confirmed in the histogram (Figure S7). However, Compound **1** was unstable with large changes in its RMSD, having an average RMSD of 5.46 Å with a standard deviation of 1.40 Å (Table 7). Compound **2** showed slight changes early in the simulation but returned to conformations similar to the docking conformation. It had an average RMSD of 1.17 Å and a standard deviation of 0.23 Å. The compound (10406567) exhibited the best stability during the MD simulation with an average RMSD of 0.96 Å and a standard deviation of 0.07 Å. This was followed by Compound **2** and 67837201; 67837201 had an average RMSD of 1.46 Å with a standard deviation of 0.10 Å. The average RMSDs for these compounds was lower than those of validamycin A and validoxylamine A, suggesting that the three compounds maintained relatively stable conformations throughout the simulation period.

#### 3.4.2. Principal Component Analyses (PCA)

**Figure 3:**
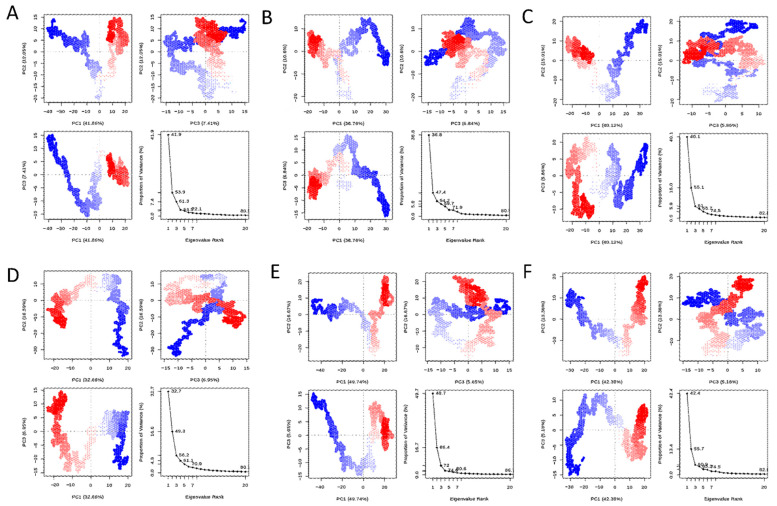
PCA plots of the protein backbone in the complex, comprising graphs of PC2 vs PC1, PC2 vs PC3, PC3 vs PC1, and an eigenvalue rank plot with the cumulative variance annotated for each data point. (**A**) Validoxylamine A; (**B**) validamycin A; (**C**) 67837201; (**D**) 10406567; (**E**) Compound **2**; (**F**) Compound **1**.

For validoxylamine A, validamycin A, 67837201, 10406567, Compounds 2 and 1, the first three principal components covered 61.3, 54.2, 61, 56.2, 72, and 60.9% of the total variance, respectively, as shown in the eigenvalue rank plots in Figure 3. This suggests that the molecular dynamics simulation procedure captured the major or dominant motions rather than the much less dominant ones. Considering the first three principal components for 67837201, 10406567, Compound **1**, and Compound **2**, the order of variance was 10406567 < Compound **1** < 67837201 < Compound **2**. PC1 accounted for 41.9, 36.8, 40.1, 32.7, 49.7, and 42.4% of the variance for validoxylamine A, validamycin A, 67837201, and 10406567, Compounds 2 and 1. When PC1 alone was considered, the order of variance was 10406567 < 67837201 < Compound **1** < Compound **2**. Of the hit compounds screened, Compound **2** had the highest variance, while 10406567 had the lowest variance either when PC1 only or the first three principal components were considered.

#### 3.4.3. Hydrogen Bond Analyses

The percentage occupancy of the h-bond >10% between the compounds and amino acid residues of the protein is presented in Table 8. For validoxylamine A, 67837201, and 10406567, Compound **1**, and Compound **2**, multiple hydrogen bonds were identified between the active site of the protein and the ligand. However, validamycin A did not form hydrogen bonds with any of the active side residues throughout the simulation. The compounds formed h-bonds with similar residues they interacted with in the molecular docking studies (either through hydrogen bond, Pi, unfavourable donor–donor, or van der Waals interactions). Compounds 1 and 2 formed hydrogen bonds with Arg195 (46.26%) and Asp150 (57.72%), respectively, which were not observed in the molecular docking studies. The hydrogen bond interaction with Asp292 was persistent for validoxylamine A (75.46%) and 10406567 (54.56%), while it had an occupancy of 22.62% for 67837201. However, it had an h bond occupancy of <10% for Compound **1** (5.92%) and 2 (6.87%). Glu258 had an h-bond occupancy of 59.33% for 67837201, Asn187 had an occupancy of 58.67% for Compound **1**, and Asp150 had an occupancy of 57.72% for Compound **2**. Hydrogen bond interactions for all the compounds with other residues were also observed, but these were not persistent (<10%) and were only present for a minority of the time-lapse of the simulation.

## 4. Discussion

In this study, validamycin A was observed to have a lower affinity for *Ag*Tre and *Aa*Tre than validoxylamine A and other previously reported compounds tested (Table 3 and Table 6). These results support previous studies in other organisms suggesting validamycin A to be a poor binder of trehalase compared to validoxylamine A. For example, Asano et al. [60] reported an IC_50_ of 72 μM for validamycin A in *Rizochtonia solani*, compared to an IC_50_ of 140 nM for validoxylamine A. Similarly, validoxylamine A has been observed to have a better inhibitory effect on trehalase than validamycin A in *Spodoptera litura* (IC_50_ of 48 nM vs. 370 nM, respectively), porcine intestine (14 nM vs. 120 nM, respectively) [61], porcine kidney (2.4 nM vs. 250 µM) [62], and termites (3.2 μM vs. 402 μM) [63]. In a study by Asano et al. [64], injection of 10 µg of validoxylamine A in the last instar larvae of *Spodoptera litura* resulted in 70% death in prepupae and 30% abnormal pupae, with no normal pupae or adult being formed. However, injection of validamycin A resulted in 25% death in prepupae, 30% abnormal pupae, and 45% normal pupae, although only 11% of the normal pupae emerged as adults [64]. These studies affirm that validoxylamine A is a better inhibitor of trehalase than validamycin A, corroborating the observation in this study. In this present study, trehazolin (binding energy of −7.5 kcal/mol) was observed to be a better binder of *Ag*Tre than validamycin A. Ando et al. [65] reported trehazolin to have an IC_50_ of 66 nM in *Rizochtonia solani* compared to IC_50_ of 72 μM for validamycin A, and 140 nM for validoxylamine A reported earlier by Asano et al. [60], thus suggesting trehazolin is a better inhibitor than validamycin A and validoxylamine A. However, in porcine kidney, Kyosseva et al. [62] reported trehazolin to have an IC_50_ of 16 nM compared to 2.4 nM for validoxylamine A and 250 µM for validamycin A. This suggests that describing trehazolin as a better trehalase inhibitor when compared to validoxylamine might be organism-dependent. In this current study, validoxylamine was observed to be the best inhibitor of *Ag*Tre among the previously reported inhibitors, followed by trehazolin. Uniflorine, trehazolamine, and casuarine had quite similar binding affinities for *Ag*Tre, while validamycin A had the least affinity (Table 3). Unlike other compounds which interacted with active site residues of *Ag*Tre, validamycin A interacted with other residues (Table 3, Figure S1). Unlike *Ag*Tre, in which validamycin A had van der Waal interactions with tyrosine 507 only in the active site, validamycin A had a carbon hydrogen bond with Arg 193, van der Waals interactions with Glu554, Lys77, and Tyr555 in the active site of *Aa*Tre. However, conventional hydrogen bonds were formed with the non-active site residues Gly192, Glu313, and Arg312. In both organisms, validamycin A had the highest binding energy among the tested ligands (Table 6). This might explain the low larvicidal activity observed with validamycin treatment in *Ae. aegypti* by Marten et al. [19]. MD simulation was employed to investigate the stability of the protein–ligand complexes in a flexible system, unlike molecular docking, which assumes that the protein and ligand are in a rigid conformation in most cases [66]. Despite the low affinity for trehalase and not interacting much with active site residues, validamycin A was relatively stable during *Ag*Tre-validamycin A MD simulation (Figure 2C, Table 7). It had an h-bond occupancy of 33.97% for Glu262 and 13.03% for Gly504 (Table 8), while the h-bonds formed with other residues persisted below 10% during the whole simulation. These results further corroborate validamycin A as a poor binder of *Ag*Tre. This suggests that these other molecules, aside from validamycin A, could serve as better insecticides for malaria vector control.

Uniflorine, the safest previously reported compound showing no predicted toxicity (Table 4), had a binding energy of −6.9 kcal/mol for *Ag*Tre and −7.6 kcal/mol for *Aa*Tre (Table 6). Uniflorine has been reported to show >5649 selectivity for *C. riparius* trehalase (177 ± 18 nM) as compared to trehalase from porcine kidney (>1 mM) [67]. Uniflorine is a weaker binder of trehalase from porcine kidney when compared to validoxylamine A, for which an IC_50_ of 2.4 nM has been reported, but a better binder than validamycin A, for which an IC_50_ of 250 μM was reported [62]. In this present study, a similar trend was observed with uniflorine having a lower affinity for *Ag*Tre and *Aa*Tre than validoxylamine A (−8.8 kcal/mol) but a better affinity than validamycin A in both organisms. Considering the high selectivity of uniflorine and its safety, it could be further tested as a possible compound for malaria vector control.

In this study, Compounds 1 and 67837201 were the hit compounds with no toxicity and yet low binding energy for *Ag*Tre (Table 3, Table 4 and Table 5). Their binding energies were higher than that of uniflorine, suggesting that they are better binders of *Ag*Tre. RMSD results from further examination of the stability of their protein–ligand complexes by MD simulation suggested that Compound **1** might have a binding energy different from that observed in the molecular docking studies as the compound was unstable during the simulation (Figure 2D). This highlights the importance of following up molecular docking studies with MD simulation, as MD simulation has been reported to help separate false positive hits from true positive hits obtained from molecular docking studies [21]. 67837201 was stable during the simulation (Figure 2C); however, unlike in *Ag*Tre, where a binding energy of −8.9 kcal/mol was observed during docking studies, it had a higher binding energy for *Aa*Tre (−5.9 kcal/mol). Both 10406567 and Compound **2** had good affinity for trehalase of both organisms (Table 6) and displayed stability throughout the simulation (Figure 2C), suggesting they could be excellent compounds for vector control. Further experimental studies to assess their toxicity are necessary, as both were predicted to have a slight probability of being nephrotoxic (Table 4). Similarly, their selectivity for these target organisms needs to be compared to that of non-target organisms in further experimental investigations. However, it has been reported that the disease arising from trehalase inhibition in humans is a mild disease, which can be reversed by avoiding the consumption of mushrooms [68], suggesting trehalase inhibitors as safer alternatives for vector control compared to, for example, organophosphates. Although trehalase enzymes are present in bacteria, fungi, and arthropods [69], uniflorine, 67837201, 10406567, and Compound **2** were predicted to be non-toxic to honeybees, crustaceans, and aquatic life. While these compounds were screened in silico and simulation performed at 310 K, reports of in vivo testing of validamycin A and validoxylamine A in different studies give confidence that the compounds identified in this study would be effective in vivo despite different climatic conditions in which mosquitoes may exist. Experimental validation remains to be carried out as the next future step.

## 5. Conclusions

This study screened trehalase inhibitors against *Ag*Tre to identify inhibitors with better affinity than validamycin A, which could be developed further for malaria vector control. The study proposes uniflorine, 67837201, 10406567, and Compound **2** as possible compounds for malaria vector control. These compounds also bind to *Aa*Tre; as such, they can be used to control *Aedes aegypti*, thus preventing transmission of several other diseases such as Mayaro and dengue fever. The trehalase inhibitors can be applied to breeding sites, as they could affect the development of mosquitoes and prevent their flight to humans, consequently preventing the transmission of mosquito-borne diseases.

## Figures and Tables

**Table 1:** Structural assessment of predicted 3D structures of *Anopheles gambiae* trehalase (*Ag*Tre).

Validation Index	SWISS-MODEL	AlphaFold	RoseTTAFold	I-TASSER	C-I-TASSER
Ramachandran Favoured (%)	94.70	96.13	98.06	77.18	79.67
Ramachandran outliers (%)	0.76	0.70	0.35	7.38	7
QMEAN	−3.05	−1.43	−0.10	−7.72	−7.20
Cβ deviations	2	1	0	61	44
Rotamers outliers (%)	0.22	0.82	0.00	14.31	14.72
Cis non-prolines	4/508	1/547	-	3/547	1/547
Twisted non-prolines	-	3/547	-	39/547	38/547
ERRAT	95.155	97.3635	96.78	90.2135	95.7721
VERIFY3D	89.81	90.18	91.23	87.02	82.28

**Table 2:** Predicted active site residues for trehalase of *An. gambiae* and *Ae. aegypti*.

Organism (Server)	Active Site Residues	Pocket Score	Probability
*An. gambiae* (CASTp)	Lys26, Arg142, Phe143, Tyr147, Trp149, Asp150, Asn186, Tyr192, Arg195, Gln197, Arg256, Glu258, Ser259, Ala287, Gly290, Asp292, Gln438, Trp439, Trp445, Tyr507, Trp515	NA ^1^ Area = 217.4 Å^2^ Volume = 116.135 Å^3^	NA
*An. gambiae* (Prankweb)	Lys26, Arg142, Phe143, Tyr147, Trp149, Asn186, Gln197, Glu258, Ser259, Ala287, Gly290, Asp292, Gln438, Trp439, Trp445, Tyr507, Trp515	37.06	0.948
*Ae. aegypti* (Prankweb)	Lys77, Arg193, Phe194, Tyr198, Trp200, Asp201, Asn237, Tyr243, Gln248, Glu309, Ser310, Ala338, Gly341, Asp343, Gln486, Trp487, Trp493, Glu539, Glu554, Tyr555, Trp563	21.72	0.855

^1^ NA: not applicable. Residues in red were not conserved as active site residues in the two organisms.

**Table 3:** Structure, chemical formula, binding energies, and binding interactions of *Ag*Tre with the control ligand, previously reported trehalase inhibitors, and the top 9 hits from the virtual screening studies.

	Pubchem ID	Structure	Chemical Formula	Binding Energy (kcal/mol)	Binding Interactions
Control ligand	443629 (Validamycin A)	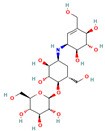	C_20_H_35_NO_13_	−5.4	Conventional hydrogen bond: Ser266, Asn307, Gly504, Glu506 Van der Waals: Tyr507, Glu508, Ser503, Gly505, Leu328, Asn436, Leu265, Glu262
Previously reported trehalase inhibitors	11450478 (Validoxylamine A)	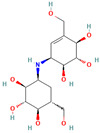	C_14_H_25_NO_8_	−8.8	Conventional hydrogen bond: Glu258, Asn186, Gln197, Asp292,Pi-Sigma: Tyr507 Alkyl and Pi-Alkyl: Phe143, Tyr147 Carbon hydrogen bond: Arg256 Van der Waals: Gln438, Ser259, Tyr192, Ala287, Arg195, Gly290, Tyr148, Trp149, Trp445, Trp515 Unfavourable Donor–Donor: Arg142, Lys26
	136245199 (Trehazolin)	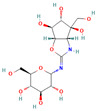	C_13_H_22_N_2_O_10_	−7.5	Conventional hydrogen bond: Glu258, Ser259, Gln197 Carbon hydrogen bond: Gly290 Van der Waals: Phe293, Arg256, Asp292, Trp439, Ala287, Trp445, Trp515, Trp149, Asn186, Asp150, Phe143, Tyr147, Tyr148, Arg195, Tyr192, Arg142, Gln438, Lys26, Tyr507
	9794258 ((-)-uniflorine A)	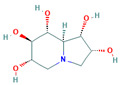	C_8_H_15_NO_5_	−6.9	Conventional hydrogen bond: Glu258, Asp292 Carbon hydrogen bond: Gln197, Gly290 Van der Waals: Phe143, Asp150, Tyr147, Asn186, Arg195, Tyr192, Ala287, Trp439, Trp445, Trp515, Trp149
	9859098 (Casuarine)	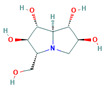	C_8_H_15_NO_5_	−6.6	Conventional hydrogen bond: Glu258, Asn186, Gln197, Asp292, Gln438 Van der Waals: Asp150, Trp149, Trp515, Gly290, Phe143, Trp439, Arg142, Ala287, Tyr192, Arg195, Tyr147, Tyr148 Unfavourable Donor–Donor: Asn186
	11148064 (Trehazolamine)	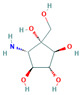	C_6_H_13_NO_5_	−6.4	Conventional hydrogen bond: Asp292 Pi-Sigma: Tyr507 Carbon hydrogen bond: Glu258 Van der Waals: Tyr147, Tyr192, Phe143, Lys26, Gln438, Ser259, Arg195, Arg256, Ala287, Trp439 Unfavourable Donor–Donor: Arg142
TOP 9 HITS	101104782	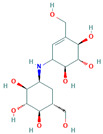	C_14_H_25_NO_8_	−9.1	Conventional hydrogen bond: Glu258, Asn186, Gln197, Asp292, Gly290, Gln438 Alkyl and Pi-Alkyl: Phe143, Lys26 Carbon hydrogen bond: Ala287 Van der Waals: Asp150, Trp149, Trp515, Tyr147, Tyr192, Arg195, Arg142, Tyr507, Trp439, Trp445, Tyr148, Ser25, Asp24, Ser259 Unfavourable Donor–Donor: Lys26
	13364642	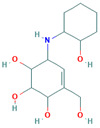	C_13_H_23_NO_5_	−9.1	Conventional hydrogen bond: Asn186 Alkyl and Pi-Alkyl: Phe143, Tyr507, Arg142 Carbon hydrogen bond: Asp292 Van der Waals: Asp150, Trp149. Trp515, Tyr147, Tyr192, Tyr507, Trp439, Trp445, Tyr148, Ser259, Ala287, Lys26, Glu258, Gly290, Gln438, Arg256 Unfavourable Donor–Donor: Gln197
	56668330	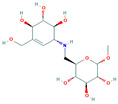	C_14_H_25_NO_9_	−9.1	Conventional hydrogen bond: Arg142 Carbon hydrogen bond: Asp292 Van der Waals: Arg256, Ser259, Tyr507, Gln438, Trp439, Phe143, Asn186, Gly290, Gln197, Trp445, Trp515, Trp149, Tyr148, Asp150, Tyr147, Ala287, Glu258, Ser151 Unfavourable Donor–Donor: Lys26
	10406567	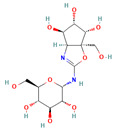	C_13_H_22_N_2_O_10_	−9	Conventional hydrogen bond: Asn186, Gln197, Asp292, Gly290, Gln438, Arg142, Tyr192 Carbon/Pi-Donor Hydrogen bond: Glu258, Ala287, Phe143 Van der Waals: Asp150, Trp149. Trp515, Tyr147, Tyr148, Arg195, Tyr507, Trp439, Trp445, Ser259, Lys26
	10690241	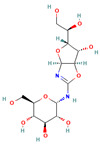	C_13_H_22_N_2_O_10_	−9	Conventional hydrogen bond: Asn186, Gln197, Asp292, Gln438, Arg142, Glu258 Pi-Sigma: Tyr507 Carbon hydrogen bond: Ala287 Van der Waals: Asp150, Gly290, Tyr192, Trp149, Trp515, Tyr147, Arg195, Trp439, Trp445, Ser259, Lys26, Phe143, Arg256
	58618560	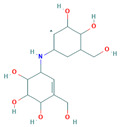	C_14_H_24_NO_7_	−9	Conventional hydrogen bond: Asn186, Gln197, Gln438, Ala287, Ser259, Trp149 Pi-Alkyl: Phe143 Carbon hydrogen bond: Ala287, Glu258 Van der Waals: Tyr147, Tyr148, Asp150, Trp515, Lys26, Tyr507, Arg142, Arg256, Asp292, Gly290, Tyr192, Arg195
	21021639	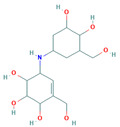	C_14_H_25_NO_7_	−8.9	Conventional hydrogen bond: Asn186, Gln438, Ala287, Ser259, Trp149, Arg142, Glu258 Pi-Alkyl: Phe143 Carbon hydrogen bond: Lys26, Glu258 Van der Waals: Gln197, Tyr147, Tyr148, Asp150, Trp515, Tyr507, Gly290, Tyr192, Ser25 Unfavourable Acceptor–Acceptor: Asp292
	25023458	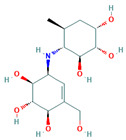	C_14_H_25_NO_7_	−8.9	Conventional hydrogen bond: Asn186, Gln197, Ser259, Glu258 Pi-Sigma: Phe143 Alkyl/Pi-Alkyl: Arg142, Lys26 Carbon hydrogen bond: Ala287, Asp292 Van der Waals: Tyr147, Arg195, Tyr192, Arg256, Tyr507, Gln438, Trp439, Gly290, Trp515, Trp149, Trp445
	67837201	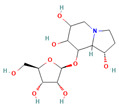	C_13_H_23_NO_8_	−8.9	Conventional hydrogen bond: Gln438, Ser259, Arg142, Lys26, Glu258 Pi-Sigma: Tyr507 Carbon hydrogen bond: Gln197, Glu258 Van der Waals: Arg256, Asp292, Ala287, Arg195, Tyr192, Asn186, Tyr147, Trp149, Gly290, Trp445, Trp515, Trp439, Phe143, Ser25 Unfavourable Acceptor–Acceptor: Glu258

**Table 4:** ADMET prediction of control ligand, previously reported trehalase inhibitors, top 9 hits, and optimised compounds.

	PubChem ID	Brenk #Alerts	Synthetic Accessibility Score	Non-Biodegradability (P) ^1^	Nephrotoxicity (P)	DILI (P)	Respiratory Toxicity (P)
Control ligand	443629	1	6.34	-	-	0.969	0.158
Previously reported trehalase inhibitors	11450478	1	5.06	-	-	0.956	0.676
	136245199	1	5.67	-	0.5267	0.195	0.126
	9794258	0	3.49	-	-	0.068	0.248
	9859098	0	3.41	0.575	0.4748	0.16	0.551
	11148064	0	3.24	0.55	0.5557	0.034	0.22
Top 9 hits	101104782	1	5.06	-	-	0.956	0.676
	13364642	1	4.6	-	-	0.835	0.917
	56668330	1	5.35	-	-	0.959	0.502
	10406567	0	5.78	-	0.4811	0.165	0.277
	10690241	0	5.82	-	0.5058	0.33	0.091
	58618560	1	4.9	-	-	0.956	0.751
	21021639	1	4.94	-	-	0.954	0.723
	25023458	1	5.02	-	-	0.958	0.81
	67837201	0	5	-	-	0.031	0.102
Optimised compounds	Compound **1**	0	5.44	-	-	0.259	0.065
	Compound **2**	0	5.11	-	0.5908	0.183	0.056

^1^ P: Probability. **#**: numbers.

**Table 5:** Structure, chemical formula, binding energies, and binding interactions of *Ag*Tre with optimised compounds.

	Pubchem ID	Structure	Chemical Formula	Binding Energy (kcal/mol)	Binding Interactions
Optimised compounds	2-{[6,7-dihydroxy-5-(hydroxymethyl)-3aH,5H,6H,7H,7aH-pyrano[2,3-d][1,3]oxazol-2-yl]amino}-6-(hydroxymethyl)oxane-3,4,5-triol (Compound **1**)	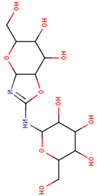	C_13_H_22_N_2_O_10_	−8.9	Conventional hydrogen bond: Lys26, Tyr507, Asn186, Trp149 Van der Waals: Gln438, Arg142, Glu258, Ser259, Arg256, Asp292, Phe293, Tyr192, Ala287, Gln197, Tyr148, Tyr147, Ser151, Asp150, Trp445, Gly290, Trp515, Trp439, Phe143
	4-(hydroxymethyl)-6-{[3,4,5-trihydroxy-6-(hydroxymethyl)oxan-2-yl]amino}-hexahydro-[1,3]dioxolo[4,5-c]pyran-2-one(Compound **2**)	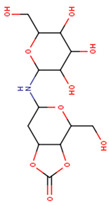	C_13_H_21_NO_10_	−8.4	Conventional hydrogen bond: Gln438, Gln197, Lys26, Pi-Sigma: Tyr507 Alkyl: Ala287 Carbon hydrogen bond: Gly290, Asp292 Van der Waals: Phe143, Trp439, Ser259, Glu258, Arg256, Tyr192, Asn186, Tyr147, Trp515, Trp445, Trp149 Unfavourable Donor–Donor: Lys26, Arg142

**Table 6:** Binding energies of compounds docked against trehalase of *An. gambiae* and *Ae. aegypti*.

	PubChem ID	Binding Energy (kcal/mol) *An. gambiae*	Binding Energy (kcal/mol) *Ae. aegypti*
Control ligand	443629	−5.4	−6.4
Previously reported trehalase inhibitors	11450478	−8.8	−8.8
	136245199	−7.5	−6.6
	9794258	−6.9	−7.6
	9859098	−6.6	−7.1
	11148064	−6.4	−6.4
Top hits	101104782	−9.1	−8.1
	10406567	−9.0	−8.3
	10690241	−9.0	−6.1
	67837201	−8.9	−5.9
Optimised hits	Compound **1**	−8.9	−6.4
	Compound **2**	−8.4	−8.4

**Table 7:** Average RMSD and standard deviation of bound protein and ligands.

Compounds	Average RMSD (SD) of Protein (Å)	Average RMSD (SD) of Ligand (Å)
Validoxylamine A	2.23 (0.43)	1.90 (0.25)
Validamycin A	1.92 (0.30)	1.95 (0.19)
67837201	2.15 (0.39)	1.46 (0.10)
10406567	2.12 (0.31)	0.96 (0.07)
Compound **1**	1.93 (0.31)	5.46 (1.40)
Compound **2**	2.38 (0.56)	1.17 (0.23)

Presented are the measured average RMSDs of the protein and ligands with their standard deviations in parenthesis after MD simulation of 1 ns.

**Table 8:** Percentage occupancy of the h-bond.

Compounds	H Bonded Amino Acid Residue (Percentage Occupancy)
Validoxylamine A	Asp292 (75.46%), Glu258 (47.48%), Tyr192 (31.81%), Gln197 (29.23%), Arg142 (19.25%), Tyr147 (12.06%), Ser259 (10.16%)—7
Validamycin A	Glu262 (33.97%), Gly504 (13.03%)—2
67837201	Glu258 (59.33%), Gly290 (45.84%), Arg142 (34.26%), Asp292 (22.62%)—4
10406567	Asp292 (54.56%), Arg195 (35.17%), Gly290 (17.67%), Glu258 (13.02%), Arg142 (25.61%), Ser259 (10.50%)—6
Compound **1**	Asn186 (58.67%), Arg195 (46.26%), Glu258 (41.12%), Lys26 (31.54%), Arg142 (30.82%), Asp150 (26.32%)−6
Compound **2**	Asp150 (57.72%), Gln438 (31.88%), Arg256 (23.38%), Arg142 (10.47%), Ser259 (10.06%)—5

The colours depict different interactions the residues formed with the active site of *Ag*Tre in the molecular docking studies. Light green: van der Waals; Red: unfavourable donor–donor interactions; Black: hydrogen bonds; Purple: Pi-interactions; Blue: does not interact in the molecular docking studies.

## Data Availability

The authors confirm that the data supporting the findings of this study are available within the article and its Supplementary Materials.

## Supplementary Materials

The following supporting information can be downloaded at: https://www.mdpi.com/article/10.3390/insects13111070/s1, Figure S1: Binding interaction of *Ag*Tre with previously reported inhibitors with docked using vina in PyRx, visualised in Discovery Studio, (**A**) residue glutamate 258, asparagine 186, glutamine 197, and aspartate 292 form conventional hydrogen bonds represented in green dotted lines with validoxylamine A; (**B**) residue serine 259, glutamate 258, and glutamine 197 form conventional hydrogen bonds represented in green dotted lines with trehazolin; (**C**) residue glutamate 258, glycine 290, glycine 197, and aspartate 292 form conventional hydrogen bonds represented in green dotted lines with uniflorine; (**D**) glutamate 258, asparagine 186, glutamine 197, glutamine 438 and aspartate 292 form conventional hydrogen bonds represented in green dotted lines with casuarine; (**E**) aspartate 292 forms conventional hydrogen bonds represented in green dotted lines with trehazolamine; (**F**) glycine 504, glutamine 506, asparagine 307, and serine 266 form conventional hydrogen bonds represented in green dotted lines with validamycin A; Figure S2: Binding interaction of *Ag*Tre with top 9 hit compounds docked using vina in PyRx, visualised in Discovery Studio, (**A**) 101104782; (**B**) 13364642; (**C**) 56668330; (**D**) 10406567; (**E**) 10690241; (*F*) 58618560; (*G*) 21021639; (*H*) 25023458; (*I*) 67837201; Figure S3: Binding interaction of *Ag*Tre with optimised compounds docked using vina in PyRx, visualised in Discovery Studio, (**A**) Compound **1**; (**B**) Compound **2**; Figure S4: Conformational changes and residue flexibilities of the Protein Cα-RMSDs during MD simulation with (**A**) validoxylamine A; (**B**) validamycin A; (**C**) 67837201; (**D**) 10406567; (**E**) Compound **2**; (**F**) Compound **1**; Figure S5: RMSD histogram of the Protein Cα-RMSDs during MD simulation with (**A**) validoxylamine A; (**B**) validamycin A; (**C**) 67837201; (**D**) 10406567; (**E**) Compound **2**; (**F**) Compound **1**; Figure S6: Ligand RMSD during MD simulation (**A**) validoxylamine A; (**B**) validamycin A; (**C**) 67837201; (**D**) 10406567; (**E**) Compound **2**; (**F**) Compound **1**; Figure S7: Ligand RMSD histogram during MD simulation (**A**) validoxylamine A; (**B**) validamycin A; (**C**) 67837201; (**D**) 10406567; (**E**) Compound **2**; (**F**) Compound **1**; Figure S8: Protein Cα-RMSFs during MD simulation with (**A**) validoxylamine A; (**B**) validamycin A; (**C**) 67837201; (**D**) 10406567; (**E**) Compound **2**; (**F**) Compound **1**.
