# Supplementary figures and images for: Anopheles gambiae Trehalase Inhibitors for Malaria Vector Control: A Molecular Docking and Molecular Dynamics Study
Source: Insects. 2022 Nov 19;13(11):1070. doi: 10.3390/insects13111070 (PMC9694508; doi:10.3390/insects13111070)

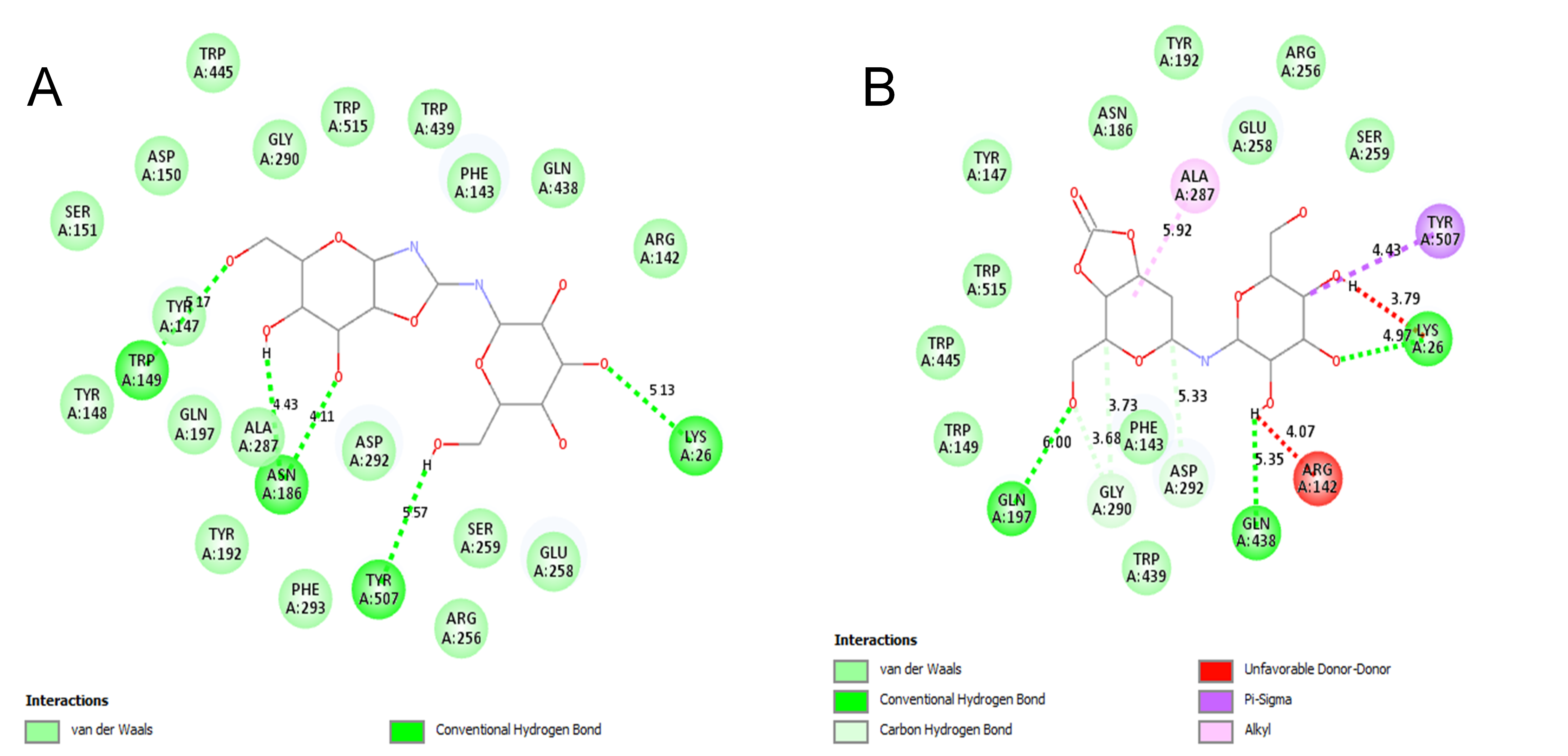

Supplement: Supplementary file 1 [file insects-13-01070-s001.zip › FIgure S3.tif]
